# Drug resistance in children at virological failure in a rural KwaZulu-Natal, South Africa, cohort

**DOI:** 10.1186/1742-6405-11-3

**Published:** 2014-01-20

**Authors:** Sureshnee Pillay, Ruth M Bland, Richard J Lessells, Justen Manasa, Tulio de Oliveira, Sivapragashini Danaviah

**Affiliations:** 1 Africa Centre for Health and Population Studies, College of Health Sciences, University of KwaZulu-Natal, Durban, South Africa; 2 Royal Hospital for Sick Children, Glasgow, UK; 3 Department of Clinical Research, London School of Hygiene and Tropical Medicine, London, UK; 4 Research Department of Infection, University College of London (UCL), London, UK; 5 Rm 135, 1st Floor, DDMRI, 719 Umbilo Road, Congella 4013, South Africa

## Abstract

**Background:**

Better understanding of drug resistance patterns in HIV-infected children on antiretroviral therapy (ART) is required to inform public health policies in high prevalence settings. The aim of this study was to characterise the acquired drug resistance in HIV-infected children failing first-line ART in a decentralised rural HIV programme.

**Methods:**

Plasma samples were collected from 101 paediatric patients (≤15 yrs of age) identified as failing ART. RNA was extracted from the plasma, reverse transcribed and a 1.3 kb region of the pol gene was amplified and sequenced using Sanger sequencing protocols. Sequences were edited in Geneious and drug resistance mutations were identified using the RegaDB and the Stanford resistance algorithms. The prevalence and frequency of mutations were analysed together with selected clinical and demographic data in STATA v11.

**Results:**

A total of 101 children were enrolled and 89 (88%) were successfully genotyped; 73 on a non-nucleoside reverse-transcriptase inhibitor (NNRTI)-based regimen and 16 on a protease inhibitor (PI)-based regimen at the time of genotyping. The majority of patients on an NNRTI regimen (80%) had both nucleoside reverse-transcriptase inhibitor (NRTI) and NNRTI resistance mutations. M184V and K103N were the most common mutations amongst children on NNRTI-based and M184V among children on PI-based regimens. 30.1% had one or more thymidine analogue mutation (TAM) and 6% had ≥3 TAMs. Only one child on a PI-based regimen harboured a major PI resistance mutation.

**Conclusions:**

Whilst the patterns of resistance were largely predictable, the few complex resistance patterns seen with NNRTI-based regimens and the absence of major PI mutations in children failing PI-based regimens suggest the need for wider access to genotypic resistance testing in this setting.

## Background

Globally and within South Africa, access to paediatric antiretroviral therapy (ART) has increased significantly and this in turn has impacted on mortality and morbidity among HIV-infected children [1-5]. The face of paediatric HIV is, thus, now that of a chronic disease rather than one that will necessarily result in death or serious morbidity [6]. The challenge of long-term adherence to ART in children gives rise to the potential for the emergence of drug resistance leading to treatment failure [7,8]. In resource–limited settings, there are challenges to the implementation, long-term effectiveness and sustainability of ART programmes where limited laboratory capacity to monitor treatment effectiveness and a lack of paediatric antiretroviral (ARV) formulations are notable restrictions [9]. There is some evidence that outcomes for children in rural areas of South Africa are poorer than those in urban areas [10]. In addition, socio-economic and psychosocial factors impact on optimal adherence and access to ART which in turn accelerate the development of drug resistance [11]. Without optimal management, patients can remain on failing regimens for long periods leading to the accumulation of drug resistance mutations, which can then confer cross-resistance to drugs in the same class and compromise future therapy [7,8]. The number of drugs available in South Africa is limited and there are currently no third-line options available to children failing therapy.

The data on ARV resistance in children in South Africa are relatively limited and have largely been restricted to urban hospital-based programmes, which may not be representative of all programmes [12-18]. Of the seven published studies in South Africa that used genotyping to determine the level of resistance the largest study included 51 genotypes and none was from a rural setting [12-18]. Continuing surveillance of drug resistance is important not only to guide paediatric ART policies but also to explore whether there might be a role for genotypic resistance testing within clinical care of HIV-infected children in this region. This is important because evidence-based management of children will ensure the longevity of their ART regimens. We have previously reported high levels of drug resistance in adults failing first-line ART in our decentralised, primary health care programme [19]. The primary objective of this study was to determine the frequency and patterns of resistance mutations in children failing first-line ART.

## Results

### Participants

**Figure 1:**
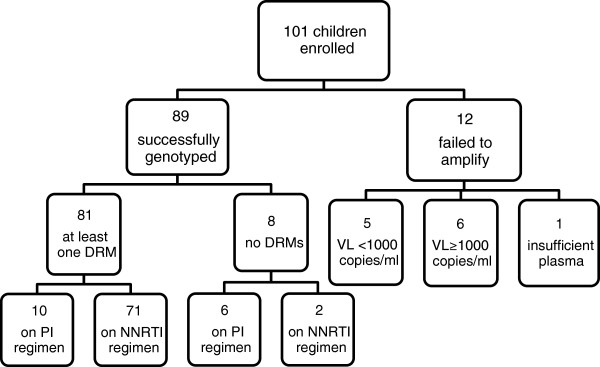
Flow chart showing children enrolled at PHC clinics and outcomes of genotyping.

Of the approximately 1653 children (≤15 years) who were initiated in and are currently active in the ART program, we identified a total of 101 children with evidence of virological failure on first-line ART and enrolled them between August 2011 and December 2012. The median time between last viral load result and genotyping was 3.1 months (IQR 1.4 - 7.0). Of the 101 samples, genotyping using the SATuRN genotyping method was successful in 89 cases (88.1%) (Figure 1). The mean viral load of the successful genotypes was 4.92 log_10_ copies/ml ± 5.3 (2.35-6.18log_10_ copies/ml). Twelve samples (11.9%) failed to amplify by PCR and were subsequently submitted for viral load quantification. Of these, six patients had a viral load at the time of genotyping of >1000copies/ml (mean = 3.77log_10_ copies/ml; range = 3.0-4.4 log_10_ copies/ml) but failed to amplify while five were suppressed with viral loads <1000copies/ml. There was insufficient sample to perform viral load quantification for one patient’s sample.

The characteristics of the children with a genotype are shown in Table 1. Sixteen children (18.0%) were on a first-line protease inhibitor-based regimen and 73 (82.0%) were on an NNRTI-based regimen at the time of genotyping. The overall median duration of ART was 3.3 years (IQR 2.5 - 4.4) and the median duration of ART failure was 1.8 years (IQR 0.8 - 2.4).

**Table 1:** Demographic and clinical characteristics of the 89 children with a genotype

**Characteristic**	**Outcome**
**Gender,** male, *n* (%)	53 (59.6)
**At ART initiation**	
**Age,** years, median (IQR)	7 (3.7-9.6)
Number of children per age group n(%)	
0-3 years	17 (23)
4-9 years	37 (50)
10-15 years	20 (27)
**Viral load**, log_10_ copies/ml, median (IQR)	4.0 (3.7-4.7)
**CD4+ cell count**, cells/μl, median (IQR)	286 (112–560)
By Age category:	
0 - 2 yrs (n = 3)	817 (31–1564)
>2 – 5 yrs (n = 29)	469 (194–619)
>5 yrs (n = 38)	200 (69–363)
**ART regimen,***n* (%)	
d4T/3TC/EFV	64 (71.9)
d4T/3TC/LPVr	12 (13.5)
ABC/3TC/EFV	8 (8.9)
ABC/3TC/LPVr	4 (4.5)
AZT/3TC/EFV	1 (1.1)
**At genotyping**	
**Age, years,** median (IQR)	10.2 (7.7 – 12.9)
Number of children per age group n(%)	
0-3 years	4 (4.5)
4-9 years	38 (42.7)
10-15 years	47 (52.8)
Viral load prior to genotyping*, log_10_ copies/ml, median (IQR)	4.2 (3.8-4.8)
CD4+ cell count prior to genotyping*, cells/μl,	460 (228–769)
median (IQR)	
By Age category:	2339 (1621–2538)
0 - 2 yrs (n = 4 )	685 (442–956)
2 – 5 yrs (n = 37)	295 (147–564)
>5 yrs (n = 47)	
**ART regimen at time of genotyping,***n* (%)	
d4T/3TC/EFV	58 (65.2)
d4T/3TC/LPVr	8 (8.9)
ABC/3TC/EFV	14 (15.7)
ABC/3TC/LPVr	8 (8.9)
AZT/3TC/EFV	1 (1.1)
**Duration of ART,** years, median (IQR)	3.3 (2.5-4.4)
**Duration of ART failure**^**†**^**,** years, median (IQR)	1.8 (0.8-2.4)
**Time between last viral load and genotype**, months, median (IQR)	3.1 (1.4-7.0)
**History of ART substitution**^**#**^**,** yes, n (%)	12 (13.5)

Key:

d4T, stavudine; 3TC, lamivudine; EFV, efavirenz; LPVr, lopinavir/ritonavir; ABC, abacavir; AZT, zidovudine; ART, antiretroviral therapy; IQR, interquartile range.

*Last measurements recorded prior to date of genotype.

^†^Duration of antiretroviral failure was estimated from the date of the first viral load >1000 copies/ml to date of genotype, unless there was a viral load <50 copies/ml in-between, in which case the time was estimated from the next viral load >1,000 copies/ml. If there was no viral load ≤1,000 copies/ml then time was calculated from date of ART initiation.

^#^Substitutions included changes of single drugs due to toxicity or guideline changes.

Of the 89 children genotyped, 41 (46.1%) had, on at least one occasion, successfully suppressed viremia to <1000 copies/ml. The median duration on ART was slightly longer for those children on an NNRTI-based regimen compared to those on a PI-based regimen (3.4 years vs. 2.7 years) but the duration of ART failure was broadly similar between the same two groups (Table 2). We noted that the clinical and demographic characteristics of patients who suppressed compared with those that never suppressed (n = 48) was comparable (Table 3). The majority of patients in both groups were on 3TC-D4T-EFV both at initiation and at the time of genotyping (Table 3). Patients who had not suppressed were on a failing regimen for a statistically shorter (p = 0.04) period of time (median = 1.6 yrs; IQR = 1.4 yrs) compared with those that did suppress viremia (median = 1.9 yrs; IQR = 1.8 yrs). In addition, children who managed to suppress their viremia during treatment were older at the time of genotyping (p = 0.02) compared with those that never suppressed (Table 3). As expected, the median viral load at the time of genotyping of patients who never suppressed (median = 4.7 log_10_ cp/ml; IQR = 0.9 log_10_ cp/ml) was significantly higher (p = 0.02) than those that did (median = 3.9 log_10_ cp/ml; IQR = 4.7 log_10_ cp/ml).

**Table 2:** Characteristics of children on NNRTI-based and PI-based regimens (based on regimen at time of genotype)

	**NNRTI (*n*** **= 73)**	**PI (*n*** **= 16)**
**At ART initiation**		
**Age,** years, median (IQR)**	7.6 (5.2-10)	1.5 (0.7-3.8)
**CD4+ cell count,** cells/μl, median (IQR)*	256 (108–477)	647 (112–817)
**Viral load,** log_10_ copies/ml, median (IQR)	3.9 (3.7-4.2)	4.7 (4.7-4.7)
**At Genotyping**		
**Age at genotyping,** years, median (IQR)	11.4 (8.9-13.4)	5.0 (2.7-7.5)
**CD4+ cell count prior to genotyping,** cells/μl, median (IQR)**	379 (167–668)	762 (603–1123)
**Viral load prior to genotyping,** log_10_ copies/ml, median (IQR)	4.3 (3.8-4.8)	4.3 (3.8-5.2)
**Comparative measures**		
**Duration of ART,** years, median (IQR)	3.4 (2.6-4.5)	2.7 (1.6-4.0)
**Duration of ART failure,** years, median (IQR)	1.8 (0.9-2.4)	1.6 (0.8- 2.3)
**Time between last viral load and genotyping,** months, median (IQR)	3.3 (1.6-7.1)	3.0 (1.1-4.85)

**Key**: Difference in means at a 1% (**) or 5% (*) level of significance using the Student’s T-Test.

**Table 3:** Comparison of outcomes for patients who successfully suppressed viremia to <1000cp/ml with patients who were unable to suppress viral replication while on treatment

	**Suppressed (n = 41)**	**Never suppressed (n = 48)**
Gender:		
Male	23	30
At initiation:		
Age, years, median (IQR)	6.9 (4.4-9.8)	7.1 (3.2-9.6)
Viral load, log_10_ cp/ml, median (IQR)	3.8 (3.4-4.7)	4.2 (3.7-4.7)
CD4, cells/μl, median (IQR)	290 (136–563)	281 (89–560)
Regimen		
3TC-D4T-EFV	33	31
3TC-D4T-LPV/r	6	6
3TC-ABC-EFV	1	7
3TC-ABC-LPV/r	0	4
3TC-AZT-EFV	1	0
At genotyping:		
Age, years, median (IQR)*	11.1 (8.5-13.1)	9.8 (5.6-12.3)

Key: *Statistical significance (T-test, P<0.05).

### Drug resistance mutations (DRM)

All of the sequences accepted for analysis were deemed of high quality having passed all quality and contamination tests as described. The HIV isolates from all patients successfully genotyped were identified as HIV-1 subtype C variants. Of the 89 genotypes, 81 (91.0%) demonstrated at least one DRM while 8 (9.0%) had no DRM (Table 4). For those on an NNRTI-based regimen, the majority of genotypes had both NRTI and NNRTI mutations. Thymidine analogue mutations (TAMs) were detected in 22 (24.7%) genotypes, while three or more TAMs were detected in only four of 89 genotypes (4.5%). The Q151M complex (a multinucleoside resistance mutation) was present in two genotypes, one from a child on an NNRTI-based regimen and one from a child on a PI-based regimen. Only one child on a PI-based regimen (1/16, 6.3%) harbored a major protease mutation and the most common pattern for children on PI-based regimens was the M184V mutation alone (Table 4).

**Table 4:** The frequency of major drug resistance mutations and resistance complexes associated with PIs, NRTIs and NNRTIs of the 89 genotyped patients

	**NNRTI-based regimen (n = 73)**		**PI-based regimen (n = 16)**	
**NNRTI mutations**	Number	Percentage	Number	Percentage
Any NNRTI DRM	60	82.2	4	25.0
L100I	5	6.9	0	0
K101EP	6	8.2	0	0
K103NRS	46	63.0	2	12.5
V106M	23	31.5	2	12.5
V108I	7	9.6	1	6.3
Y181C	2	2.7	0	0
Y188HCL	7	9.6	1	6.3
G190AS	9	12.3	1	6.3
P225H	14	19.2	0	0
M230L	3	4.1	0	0
**NRTI mutations**				
Any mutation	63	86.3	10	62.5
M41L	7	9.6	0	0
K65NR	4	5.5	1	6.3
D67NG	8	11.0	0	0
K70ER	7	9.6	0	0
L74VI	4	5.5	1	6.3
Y115F	3	4.1	2	12.5
M184VI	60	82.2	10	62.5
L210W	1	1.4	0	0
T215FYI	9	12.3	0	0
K219QREN	5	6.9	0	0
Any TAMS	22	30.1	0	0
1 TAM	11	15.1	0	0
2 TAMs	7	9.6	0	0
≥3 TAMs	4	5.5	0	0
Q151M complex	1	1.4	1	6.3
**PI mutations**				
Any PI mutation	0	0	1	6.3
V82A	0	0	1	6.3

An analysis of the genotypic susceptibility scores (GSS) of patients revealed that the median GSS for patients on a PI regimen (median = 2.0, IQR = 1.25) was higher as compared with those on an NNRTI-regimen (median = 1.0, IQR = 0.5). The majority (n = 12, 75%) of the patients on a PI-regimen, had GSS scores ≥2 while only 10 (14%) of those on an NNRTI-based regimen had comparable scores.

## Discussion

In this study, we determined the frequency and patterns of drug resistance mutations in children failing first-line ART in a rural primary health care ART programme where care is delivered largely by nurses and counselors. For older children on NNRTI-based regimens, drug resistance mutations were detected in the majority. Whilst in most cases the mutations would be unlikely to significantly compromise a second-line regimen based on a ritonavir-boosted PI, five patients had complex mutation patterns (three or more TAMs or Q151M complex) that might substantially limit the future activity of the NRTI class of drugs. In contrast, the younger children on PI-based regimens more often had no drug resistance mutations (six of 16 cases) and all but one had an absence of major protease mutations. This suggests a potential need for drug resistance genotyping, particularly in this group on PI-based regimens, to determine the appropriateness of regimen switch and to preserve first-line regimens where possible.

These data represent one of the largest drug resistance studies of paediatric patients failing ART undertaken thus far in South Africa [12-18]. In addition, this study was unique in being from a rural decentralized primary health care programme where the delivery of ART to adults and children has scaled up rapidly. The characteristics of the children included in the study highlight some of the challenges of ART delivery in this setting, with very long time spent on failing regimens, a finding also reported amongst our adults on ART [19]. This suggests not only challenges to long-term adherence but also deficiencies in following the protocol for virological monitoring and switch to second-line ART guidelines. Problems with delayed switching have been well documented in South Africa [1,9,17] and these may be particularly problematic with children as many nurses and counselors are not confident managing paediatric ART and receive insufficient support and training in these issues. Some of the barriers to adherence in this setting are complex and difficult to address with the resources available within the health system. Furthermore, in rural areas with largely paper-based systems, results may simply be lost or misfiled and therefore overlooked.

First-line ART in South Africa using NNRTI-based regimens is challenging given the low genetic barrier of currently available drug options such as efavirenz and nevirapine and the fact that only one or two key DRMs are required to confer high-level resistance or cross-resistance to a drug class [20]. High-level resistance mutations and cross-resistance severely compromises future ART options, a dire consequence for paediatric patients who require lifelong ART. We detected high levels of drug resistant mutations in the group failing NNRTI-based regimens. The majority (80%) had both NRTI and NNRTI resistance mutations. The proportion that had complex NRTI resistance patterns, such as three or more TAMs or Q151M complex, was lower (5.5%) than another recent study of 38 children on ART from the same province where 39% had three or more TAMs [17] despite a shorter duration (median = 2.8 yrs; IQR = 1.9- 2.3 yrs) on ART compared with our cohort (median = 3.3 yrs; IQR = 2.5-4.4 yrs). This suggests that the majority of children failing first-line NNRTI-based regimens in our cohort should retain susceptibility to a second-line regimen consisting of two alternative NRTIs and a ritonavir-boosted PI.

It was noteworthy that only one of 17 children on a PI-based regimens had a major PI resistance mutation. The low prevalence of PI mutations has been previously described in a number of manuscripts [17,21,22] This does raise the possibility of differential adherence to different components of the ART regimen (lopinavir/ritonavir syrup can be poorly tolerated), to problems with dosing of lopinavir/ritonavir syrup or possibly to drug-drug interactions particularly for those co-infected with TB, all issues we were unable to explore in detail for this study but which are subject to on-going research.

The finding that around nine in ten children with virological failure had at least one drug resistance mutation is consistent with other studies from South Africa [22-24] and a systematic review of studies from low- and middle-income countries, which reported a pooled proportion of 90% of children on ART with any DRM [8] The proportion with TAMs (23%) was similar to that seen in a similar paediatric programme in the Western Cape (19%) [22], although lower than the 56% reported in a systematic review of first-line failure of paediatric patients [8]. The proportion with TAMs was surprisingly low given the long duration of virological failure, and was also lower than the 40% reported from adults in our programme with a similar duration of ART and similar time on a failing regimen in an adult cohort from the same region [19]. This might suggest that adherence levels were either too low or too variable for the accumulation of TAMs over time. Alternatively, there was differential adherence to components of the regimen, with avoidance or suboptimal dosing of stavudine (d4T). The lack of major PI mutations in the young children on LPV/r-based regimens is consistent with other studies from the region which have shown PI mutations to be much more commonly associated with full dose ritonavir-based regimens [16,21-23].

Currently our genotyping costs are approximately 50 US$ at reagents cost and less than 100US$ when staff and transport costs are added on. The normal cost of genotyping is 250–300 US$ in the public sector. In order to facilitate large-scale genotyping and in the interest of reducing costs, we did not perform a pre-genotype confirmatory viral load, yet we successfully genotyped 88% (89/101) of our cohort. Our genotyping system and reagents are likely to be affordable to upper middle-income countries like South Africa and Botswana but further cost reductions would be required to make drug resistance testing affordable in lower middle-income and low income countries within Africa. An additional feature of our study was that genotypes directed subsequent clinical care where a doctor, social worker and other clinic staff managed patients from enrolment to implementing an intervention post-genotyping as was carried out in an adult cohort from the same area. The results from our study addressed three of the ten goals of the Department of Health 2010 ART Guidelines in that we present a means of achieving the best outcomes for HIV-infected patients receiving ART in a cost-effective manner; we employ existing infrastructure, that of a decentralised rural public health clinic facility for patient management; and by identifying DRMs early we ensure patient retention on lifelong ART by instituting early interventions to halt ART failure and prevent morbidity and mortality.

Interpretation of these data should be subject to some limitations of the study. This was a cross-sectional study and whilst we identified as many children with first-line ART failure as possible, we were unable to accurately estimate what proportion of all children on ART had virological failure and we cannot be certain that we included all children meeting the eligibility criteria. Further, we did not have accurate information on prior exposure to pMTCT regimens either in the mother or infant therefore have no means of assessing its impact on the spectrum of DRMs we observed in this cohort. We can speculate that the patterns of NNRTI mutations we observed are suggestive of and may arise from pMTCT exposure, however we cannot determine with certainty from our data whether DRMs in these children were acquired or transmitted via MTCT. We note this as a limitation of the present study. Although these data represent the largest group of genotypes for children failing first-line ART in South Africa, the numbers remain small. This highlights the need for collaborative studies and surveillance from multiple representative sites in order to inform national policies. The data regarding protease inhibitor resistance mutations could be limited by exploring only the protease gene and it is possible that we missed mutations at other sites, e.g. Gag cleavage sites [25].

## Conclusions

In conclusion, this study has demonstrated that the spectrum of drug resistance mutations in this rural cohort is varied. The concerning prevalence of high-level resistance mutations particularly the frequency of TAMs amongst this paediatric cohort is an indicator of the time these patients spent on a failing regimen. This highlights the need for timely identification of patients failing ART and the implementation of early interventions be it drug switches or effective, reinforced, adherence counseling with appropriate follow-up. The use of genotyping, without a concurrent viral load, was efficient at identifying patients failing as a result of resistance mutations, determining the resistance profile of the patients and directing interventions for patient management. In this way, treatment options can be extended, a critical consideration for paediatric patients.

## Methods

### Setting

The study was conducted in the predominantly rural Hlabisa health sub-district in northern KwaZulu-Natal, South Africa. The programme, delivered by the Department of Health with support from the Africa Centre for Health and Population Studies (http://www.africacentre.ac.za), has been described previously [1,23,26]. HIV treatment and care is fully devolved to 17 primary health care (PHC) clinics and delivered largely by nurses and counsellors, with medical officers visiting clinics on a weekly or fortnightly basis. The programme adheres to national ART guidelines. At the time of the study, children eligible for ART aged 0–3 years (or weight under 10 kg) were commenced on a protease inhibitor (PI)-based regimen whereas children older than 3 years were commenced on a non-nucleoside reverse-transcriptase inhibitor (NNRTI)-based regimen. Viral load monitoring was scheduled six-monthly, although precise timing of viral load measurements was quite variable. Treatment of HIV-infected children was scaled up from late 2004 but accelerated in 2008 [1,23,27].

### Study design

This cross-sectional study enrolled children (≤ 15 years of age) who had been receiving ART for more than 12 months with evidence of virological failure, defined as two consecutive viral loads >1000 copies/ml. Children with virological failure were identified from all 17 clinics both passively, during routine clinic visits, and actively, through interrogation of the programme database, which is housed at the Africa Centre and also by looking at all paediatric case files at the clinics. At the same time, the files of any children not eligible for the study but who were overdue for a routine viral load measurement were noted and a blood sample, to be taken at the next clinic visit, was requested. Single drug substitutions were allowed for toxicity or in the event of guideline changes. Any child meeting the enrolment criteria was subsequently enrolled. A medical officer enrolled all children and, either a parent or guardian provided the written informed consent. Demographic and clinical information were collected from clinical charts at the time of enrolment and a venous blood sample (4 ml EDTA tube) was collected on the same day.

### Laboratory methods

Blood specimens, collected at the primary health care clinics, were transported from the Africa Centre to the Durban laboratory (~200 km away) on the same day of collection. Samples were received at the laboratory, recorded in a Laboratory Information Management System (LIMS) and the plasma was harvested and stored at -80°C until use. This blood specimen was collected specifically for resistance genotyping and was not reserved for viral load testing.

We used the affordable and open access Southern African Treatment Resistance Network (SATuRN) drug resistance genotyping system [Manasa et al. in preparation]. Briefly, this is an in-house method that costs approximately 50 US$ at reagent price. In order to keep the costs low, no viral load was performed before drug resistance genotyping. Ribonucleic acid (RNA) was extracted using the Qiagen RNA Mini kit (Qiagen N.V., Venlo, Netherlands) and reverse transcribed using the Superscript® III First-Strand Synthesis kit (Life Technologies, Carlsbad, CA) and RT21 specific primer (CTGTATTTCAGCTATCAAGTCCTTTGATGGG). A 1315 bp fragment of the *pol* gene, covering all the 99 protease codons and the first 240 codons of the reverse transcriptase (RT) gene was amplified with the high fidelity proofreading Platinum® Taq DNA polymerase (Life Technologies, Carlsbad, CA) and primers MAW26(TTGGAAATGTGGAAAGGAAGGAC) and RT21 (CTGTATTTCAGCTATCAAGTCCTTTGATGGG) for the first step and PRO1 (TAGAGCCAACAGCCCCACCA) and RT20(CTGCCAATTCTAATTCTGCTTC) for the second step polymerase chain reaction (PCR). Population sequencing was performed on successfully amplified PCR products that were identified by gel electrophoresis and visualized as a 1.3 kb fragment under ultraviolet (UV) light. The PCR products were cleaned using the PureLink® PCR purification kit (Life Technologies, Carlsbad, CA) and were sequenced using the Sanger BigDye® terminator sequencing protocol (Life Technologies, Carlsbad, CA) and four bidirectional primers (RTC1F-ACCTACACCTGTCAACATAATTG, RTC2R-TGTCAATGGCCATTGTTTAACCTTTGG, RTC3F-CACCAGGGATTAGATATCAATATAATGTGC, and RTC4R-CTAAATCAGATCCTACATACAAGTCATCC), and run on an ABI 3130xl genetic analyser (Life Technologies, Carlsbad, CA), finally generating a consensus sequence spanning 300 amino acids of RT and 99 amino acids of the protease gene. The first 240 codons of the RT gene cover all currently recognized RT mutations associated with resistance to available RT inhibitors. Samples that failed to amplify were submitted for viral load testing using the Biocentric HIV RNA Charge Virale method (Biocentric, Bandol, France) with a detection limit of 50 copies/ml.

### Sequence assembly and quality analysis

Sequences were imported into Geneious (Biomatters Ltd, Auckland, New Zealand), visually edited and deemed high quality if the quality score was higher than 80% post-trimming. Once trimmed these fragments were mapped to a reference strain (Accession# JN665021.1) to create a contig, which was further assessed for quality and ambiguities before a consensus sequence was extracted. Prior to the detection of drug resistance mutations (DRM) using bioinformatics software applications, we submitted each consensus to the Calibrated Population Resistance Tool (CPR) (http://hivdb.stanford.edu) for a final quality check. Sequences were deemed high quality if they had no ambiguities or insertions/deletions. In order to test for contamination we blasted our sequences against the public dataset using NCBI blast (http://www.ncbi.nlm.nih.gov/blast) and against our local database using a BlastServer application. Sequences were deemed not a contaminant if the identity to previously genotyped samples was lower than 98%. Finally, we constructed a maximum likelihood (ML) phylogenetic tree using phyML with GTR, gamma and percentage of invariable sites estimated from dataset. Trees were evaluated with 100 bootstraps and visualized in FigTree (http://tree.bio.ed.ac.uk/software/figtree/) in order to identify contamination where sequences cluster together with low genetic diversity.

### Data analysis

All sequences, laboratory results (viral loads, CD4+ cell counts) and clinical and demographic data were entered into the SATuRN RegaDB database [28,29]. The database first identifies the subtype of each sequence using Rega HIV Subtyping tool version 2 [30,31], and thereafter determines the effect of DRMs on antiretroviral susceptibility using the three primary drug resistance interpretation algorithms, developed at Stanford University, Agence nationale de recherches sur le sida et les hépatitis virales (ANRS) and the Rega Institute. The amino acid positions on the RT gene relevant to the NRTI drug resistance mutations were 41, 62, 65, 67, 69, 70, 74, 75, 77, 115, 116, 151, 184, 210, 215 and 219. For the NNRTI drug class we included the amino acid positions 100, 101, 103, 106, 108, 138, 179, 181, 188, 190, 221, 225, 227 and 230 of the RT gene. Finally, for the PI drug class we interrogated our sequences using the following major drug resistance amino acid positions of the protease gene: 30, 32, 46, 47, 48, 50, 54, 58, 74, 76, 82, 83, 84, 88 and 90. Mutations were coded as DRMs at these positions based on the IAS mutation list of 2013 [32]. Drug resistance mutations, clinical measurements and demographic data were exported from RegaDB for analysis.

Descriptive statistics were used to summarise the demographic and clinical characteristics. Medians and the interquartile range (IQR) were calculated for continuous data. For analysis of drug resistance mutations, frequency distributions of the major DRMs were derived. Duration on ART was defined as the number of months between the date of ART initiation and the date of genotyping. The “duration of ART failure” was defined as the period from the date of the first viral load >1000 copies/ml to date of genotyping, unless there was a viral load <50 copies/ml in between, in which cases the duration was estimated from the time of the next available viral load >1000 copies/ml. If there was no viral load ≤1000 copies/ml then duration was calculated from date of ART initiation.

### Ethics approval

The study was approved by the Biomedical Research Ethics Committee of the University of KwaZulu-Natal (ref. BF052/10) and the Health Research Committee of the KwaZulu-Natal Department of Health (HRKM 176/10).

## Abbreviations

ART: Antiretroviral therapy; ARV: Anti-retroviral; CPR: Calibrated population resistance; DRMs: Drug resistance mutations; HIV: Human immunodeficiency virus; LIMS: Laboratory information management system; NRTI: Nucleoside reverse transcriptase inhibitors; NNRTI: Non-nucleoside reverse transcriptase inhibitors; PCR: Polymerase chain reaction; PI: Protease inhibitors; SATuRN: Southern African Treatment Resistance Network; TAMs: Thymidine analogue mutations.

## Competing interests

The authors declare no competing interests in relation to this manuscript.

## Authors’ contributions

SP and JM carried out the laboratory assays. SD and JM performed sequence analysis. SD performed the statistical analysis. SP and SD produced the first draft of the manuscript. TdO, RMB, RJL and SD formulated the design and coordinated the study. All authors contributed to the editing and production of the final manuscript. All authors read and approved the final manuscript.

## Data and materials availability

Sequences generated for this analysis are available on request from the authors.
